# P-Glycoprotein-Activity Measurements in Multidrug Resistant Cell Lines: Single-Cell versus Single-Well Population Fluorescence Methods

**DOI:** 10.1155/2013/676845

**Published:** 2013-11-17

**Authors:** Jennifer Pasquier, Damien Rioult, Nadine Abu-Kaoud, Sabine Marie, Arash Rafii, Bella S. Guerrouahen, Frank Le Foll

**Affiliations:** ^1^Laboratory of Ecotoxicology UPRES EA 3222, IFRMP 23, University of Le Havre, 76058 Le Havre Cedex, France; ^2^Department of Genetic Medicine, Weill Cornell Medical College, New York, NY 10022, USA; ^3^Stem Cell and Microenvironment Laboratory, Weill Cornell Medical College in Qatar, Doha 24144, Qatar

## Abstract

*Background*. P-gp expression has been linked to the efflux of chemotherapeutic drugs in human cancers leading to multidrug resistance. Fluorescence techniques have been widely applied to measure the P-gp activity. In this paper, there is a comparison between the advantages of two fluorescence approaches of commonly available and affordable instruments: the microplate reader (MPR) and the flow cytometer to detect the P-gp efflux activity using calcein-AM. *Results*. The selectivity, sensibility, and reproducibility of the two methods have been defined. Our results showed that the MPR is more powerful for the detection of small inhibition, whereas the flow cytometry method is more reliable at higher concentrations of the inhibitors. We showed that to determine precisely the inhibition efficacy the flow cytometry is better; hence, to get the correct *E*_max_ and EC_50_ values, we cannot only rely on the MPR. *Conclusion*. Both techniques can potentially be used extensively in the pharmaceutical industry for high-throughput drug screening and in biology laboratories for academic research, monitoring the P-gp efflux in specific assays.

## 1. Background

Development of chemoresistance by cancer cells is a major burden in cancer treatment. Tumor resistance to specific treatment could result from (i) interaction with the host [1] or (ii) genetic or epigenetic alterations of malignant cells [2]. Resistance may be innated or acquired during treatment. In some patients, prolonged exposure to a single agent may lead to resistance to multiple other structurally unrelated antineoplasic drugs, a phenotype early defined as Multidrug Resistance (MDR) [3]. MDR has been closely related to overexpression of a membrane-associated 170-kD transmembrane glycoprotein, P-glycoprotein (P-gp), which appears to play a key role in drug efflux [4, 5]. P-gp is a member of a highly conserved superfamily of ATP-binding cassette (ABC) transport proteins that actively pump out of the cell many potentially endogenous and exogenous toxic compounds [6–8], collectively called P-gp allocrites [9]. P-glycoprotein is therefore considered as a biomarker for drug resistance [10]. Detection of P-gp-positive cells within tumors have been carried out by various techniques [11] such as determination of ABCB1 transcript levels by RT-PCR [12] and protein expression by immunocytochemistry [13]. However, a better correlation between cell resistance factor and presence of P-gp was obtained in functional assays where cell efflux capability is quantified by using a P-gp fluorescent allocrite [14, 15]. Direct measurement of P-gp activity offers a better level of functional integration, taking into account posttranscriptional and extragenetic regulations of efflux-based resistance to cytotoxics [16]. It then becomes mandatory to accurately evaluate P-gp activity in order to assess its role in the occurrence of chemoresistance.

Calcein acetoxymethyl ester (calcein-AM) is a nonfluorescent, highly lipid soluble dye that passively crosses plasma membranes. Calcein-AM is widely used to quantify MDR efflux activity, as this molecule behaves as an allocrite for several ABC transporters, namely, ABCB/P-gp and ABCC/MRP (Multidrug Related Protein) pumps [17, 18]. Once inside the cell, ester bonds are cleaved by endogenous esterase, transforming calcein-AM into a hydrophilic and intensely fluorescent calcein, which is well retained in the cytosol. MDR cells expressing high levels of P-gp rapidly extrude nonfluorescent calcein-AM from the plasma membrane, reducing accumulation of fluorescent calcein in the cytosol. As a result, fluorescence intensity is inversely related to P-gp activity.

The calcein fluorescence level can be measured by a fluorometer, often a microplate reader (MPR), assessing the fluorescence as a whole (cultures isolated from biological samples) [19] or cell by cell using a flow cytometer [20]. However, influence of the measuring approach onto the deduced mean cell fluorescence or onto the reconstituted cell fluorescence distribution in the analyzed population is often not considered. Herein, we have compared well-fluorescence and cell-fluorescence methods in drug-resistant human MCF-7 and Hs578T cell lines in order to understand better how variations of cell fluorescence intensity can affect the signals given by the instruments.

## 2. Results

**Figure 1:**
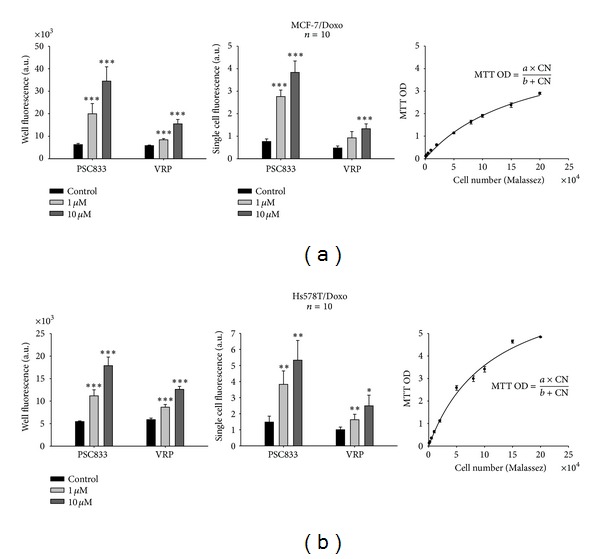
Effects of P-glycoprotein blockers on calcein-AM efflux obtained by quantifying whole well or cell-normalized well fluorescence with a microplate reader. P-gp activity was measured as the ability to efflux the fluorescent P-gp allocrite calcein-AM. MCF-7/Doxo (a) or Hs578T/Doxo (b) multidrug resistant P-gp overexpressing breast cancer cell lines were incubated with 0, 1, or 10 *μ*M of the P-gp antagonists verapamil (VRP) or PSC833. Left: global cell population P-gp activity was quantified by measuring whole well fluorescence (arbitrary units, A.U.) in 6-plicates for 10 repeated experiments in each condition. Middle: a normalized single cell P-gp activity (A.U.) was obtained from cell population in each well by computing the ratio of whole well fluorescence to the number of cells in the corresponding well. In this respect, an MTT assay was systematically performed after the fluorescence readings to determine the number of cells in each well of the microplate. Right: MTT calibration curves. MTT optical density (MTT OD) varied as a function of the number of cells (NC) deposited in the wells, in a saturation type Mickaelis-Menten relationship. For calibration, the number of cells was determined by using a Malassez counting chamber. Curve fitting to the data gave the following parameters: *a* = 5,27; *b* = 175092 for MCF-7/Doxo cells and *a* = 7,64; *b* = 113025 for Hs578T/Doxo cells, respectively. Data are presented as mean ± sem with *n* = 10 independent assays per data point. Results significantly different from the control are indicated (*P < 0,05; **P < 0,01; ***P < 0,001; paired Student's *t*-test).

The P-glycoprotein activity was assessed by using fluorigenic dye calcein-AM, as a substrate for efflux of P-gp. As a result, calcein fluorescence is inversely proportional to P-gp activity. Two methods (flow cytometry and microplate reader) have been optimized to measure the effect of a competitive (VRP) and a noncompetitive (PSC833) P-gp inhibitor on the calcein accumulation in two parental breast cancer cells lines, and their resistant variants MCF-7/Doxo (Figure 1(a)) and Hs578T/Doxo (Figure 1(b)). In this study we compared these two approaches in order to assess their sensitivity and specificity for P-gp detection in accumulation and retention assays. Ten experiments have been performed for each condition. One experiment consists in 6 replicates in microplate reader and a sample of 10,000 cells using a flow cytometer.

### 2.1. Quantifications of the Calcein AM Fluorescence in a Single Well Microplate Reader versus Single Cell

First, we determined with microplate reader the effect of VRP and PSC833 P-gp inhibitor on the intracellular trapping of calcein (Figure 1). Then, we adapted the microplate reader method with few modifications to increase its sensitivity. This gives us a global fluorescence of the well without taking into account the cell number. To overcome this issue, we decided to carry out MTT assays, to take into consideration the heterogeneity in the cell number distribution while setting up the experiment. We generated an OD/MTT scale by reading a known number of cells after MTT (*n* = 6). We generated an MTT calibration curve for each cell line which can fit by a classical enzymatic curve (MCF-7/Doxo *r* ^2^ = 0.9773; Hs578T/Doxo *r* ^2^ = 0.9840). MTT OD was between 0.2 and 0.8, consequently in the linear part of the curve, allowing us to determine the cell number. It is interesting to note that the cells number gave an ODmax/2 at a value of 175000 cells/well for MCF-7 compared to 115000 cells/well for Hs578T. This suggests that for comparing both cell lines, it is important to take into account an activity/number of cells and not an activity/OD MTT. Therefore, the single cell fluorescence was obtained after normalization with the cell number in each well. Applying this method of analysis, we have been able to express microplate reader calcein fluorescence in single cell fluorescence. Interestingly, it has been observed that the PSC833 is more effective than the VRP.

### 2.2. Flow Cytometry Cell Population Fluorescence (FL1) versus Fluorescence Concentration (FL1-FC)

**Figure 2:**
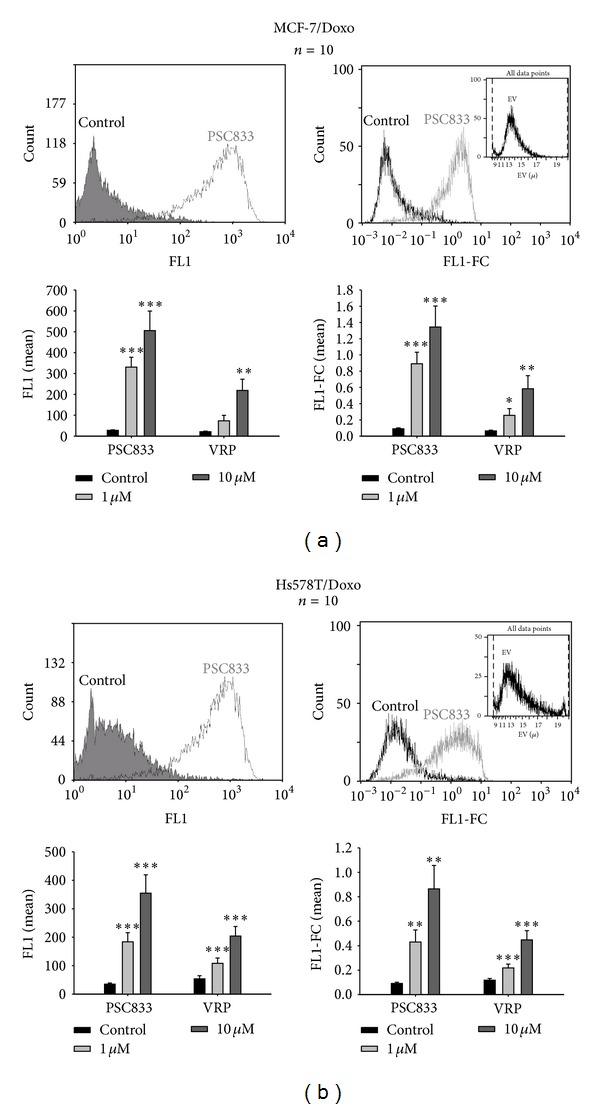
Effects of P-glycoprotein blockers on calcein-AM efflux obtained by analyzing single cell fluorescence (FL1) or fluorescence concentration (FL1-FC) with a flow cytometer. P-gp activity was followed with calcein-AM as a fluorescent probe. In each flow cytometry measurement, a sample of 10 000 cells was analyzed. (a) Top panels, left: superimposed all-events histograms of calcein fluorescence distribution (log scale) in control MCF-7/Doxo (solid gray histogram) and MCF-7/Doxo preincubated with the P-gp noncompetitive antagonist PSC-833 (10 *μ*M, open histogram). Right: the amount of fluorescence per cell is expressed as FL1-FC (fluorescent light in channel 1-fluorescence concentration) which is the fluorescent light (FL) divided by the electronic volume (EV) determined by the flow cytometer according to the Coulter Principle. The EV distribution of the sample is given in insert. Bottom panels: the two histograms present the mean fluorescence, FL1 (left) and the cell volume normalized fluorescence, FL1-FC (right) without or with 1 *μ*M or 10 *μ*M of PSC833 or verapamil (VRP) for 10 repeated experiments. (b) The same experiments were carried out in Hs578T/Doxo. Data are presented as mean ± sem with *n* = 10 independent assays per data point. *P < 0,05, **P < 0,01, ***P < 0,001.

By using a Beckman Coulter cell lab quanta with electronic volume instead of forward scatter (Figure 2), we were able to take into account the cell volume in fluorescence analysis. This normalization is important as the cell volume is changing among the same population. Therefore, we determined both the specific cell fluorescence concentration (FL1-FC) and the classic fluorescence (FL1). The results are reported in Figure 2. Then we measured the effect of the two P-gp blockers. No significant differences between analyses on FL1 or FL1-FC have been found. For further experiments, we choose to read our sample in FL1-FC. Indeed, the specific cell fluorescence concentration can potentially be used with primary cultures that are known to be very heterogeneous. We confirmed by this method that the PSC833 is more effective than the VRP.

### 2.3. Limit of Detection (LOD)

We wanted to determine the LOD of both methods used to monitor the P-gp efflux activity under treatment. The LOD of an analytical method is an important parameter when quantitative measurements have been done. In quantum chemistry, LOD is defined as the smallest concentration of a substance that could be detected but not quantified. The LOD of our methods correspond to the smallest efflux activity variation measurable between two P-gp inhibitors. Therefore, we choose a low concentration of inhibitors 100 nM of PSC833 or VRP. The intermethods comparison indicates that for the less powerful blocker, VRP, the LOD was enhanced using the microplate reader. However, for the most powerful, PSC833, the LOD is similar using both techniques. For the MPR, the intramethod comparisons compare the mean fluorescence versus the normalize fluorescence; and for the flow cytometry, to compare FL1 to FL1-FC. In both cases, LODs were not enhanced with the different data processing. A summary of our results is shown in Table 1. 

### 2.4. Linearity

**Figure 3:**
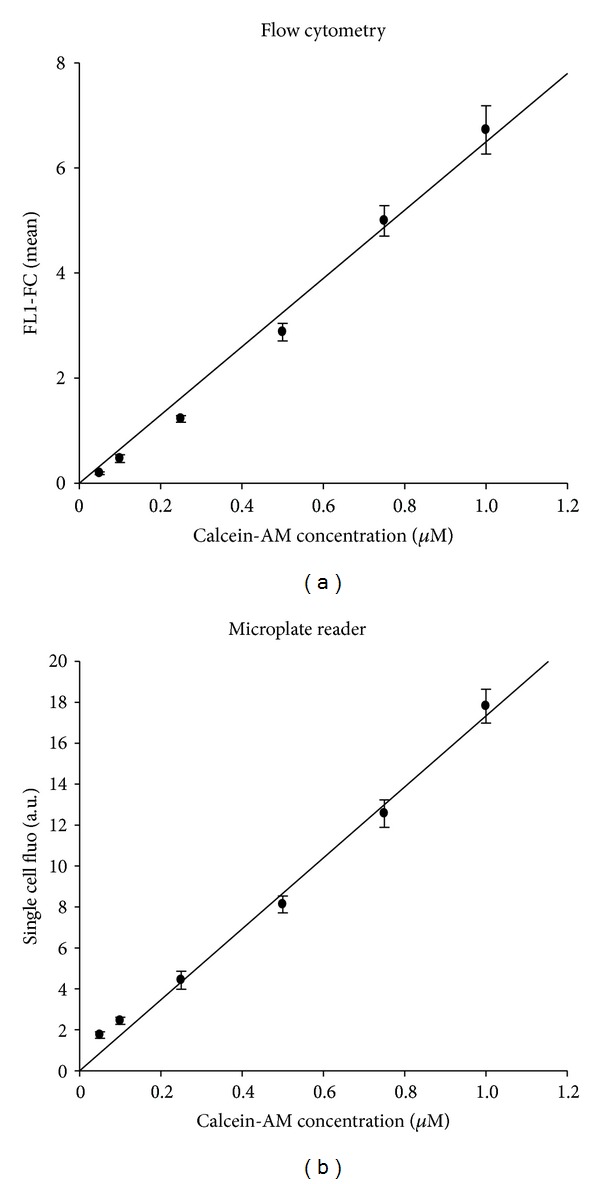
Microplate reader and flow cytometer fluorescence responses linearity. MCF-7 drug-sensitive parental cells were incubated during 15 minutes in the dark with 0,05; 0,1; 0,25; 0,5; 0,75, or 1 *μ*M of calcein-AM. Cells were analysed by flow cytometry for mean volume-normalized fluorescence (FL1-FC, (a)) and by a microplate reader for cell-normalized well fluorescence (b). Data are presented as mean ± sem with *n* = 10 independent assays per data point.

Linearity defines the analytical response as a function of analyte concentration over which acceptable linearity is achieved. Therefore, MCF-7 parental sensitive cells were incubated with different concentrations of calcein-AM (Figure 3). Linear regression has been used to fit each concentration (microplate reader *r* ^2^ = 0,9929; flow cytometry *r* ^2^ = 0,9951). Measured values were proportional to calcein concentration used. Hence, we can conclude that linearity is acceptable up to 1 *μ*M. For monitoring P-gp efflux, we used a concentration of 0.25 *μ*M; the linearity was therefore adequate for both methods.

### 2.5. Precision

The precision (also called reproducibility or repeatability) is the degree to which repeated measurements are able to show the same results under unchanged conditions. Thus, precision quantifies the variability of an analytical result as a function of operator, method manipulations, and day-to-day environment. Statistical analysis of data generated are essential to demonstrate assay precision.

**Figure 4:**
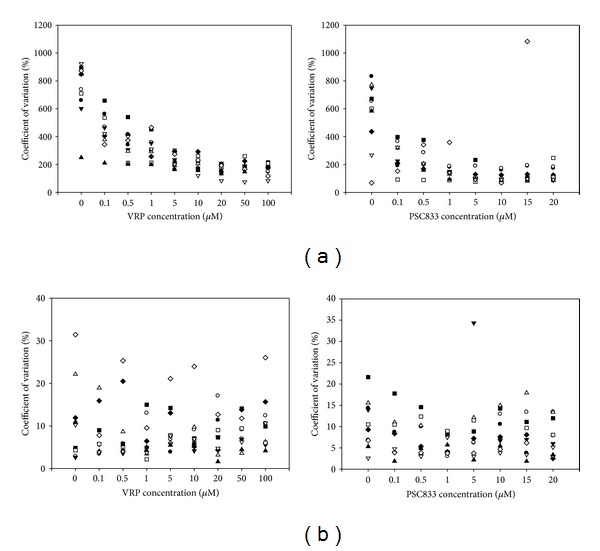
Intramethod technical replicability within ten independent biological samples. (a) Graphs show coefficient of variation (CV) of ten independent samples analyzed by flow cytometry after treatment with various concentrations of VRP (*left*) or *PSC833 (right*). A sample consists in the analysis of 10 000 cells. CV is expressed as the ratio of the standard deviation (SD) of the peak to the mean channel value (in percent) and provides a measure of the variability in signal intensity. (b) Coefficient of variation (CV = 100 × SD/mean) of 6-plicated measurements of fluorescence obtained with a microplate reader for ten independent biological samples exposed to different concentrations of VRP.

Repeatability, in flow cytometry, consists to analyse the peak distribution in only one experimentation. The coefficient of variation (CV) obtained from the 10 experiments with each blocker has been determined using the cell lab quanta software (Figure 4(a)). In the control conditions (concentration = 0), the CV values are scattered. This means that the efflux capacity of the P-gp is heterogeneous in resistant cell population. It has been observed that CV values decrease as VRP or PSC833 concentration increases. All the cells converge to a nonactivity response corresponding to the concentration maximal for the calcein in the cells. Moreover, the CV values distribution becomes more clustered when the blocker concentration increases. In conclusion, there is a better repeatability with higher P-gp blocker concentrations.

Using the microplate reader, repeatability corresponds to the CV values of 6 replicates carried out for each experiment (Figure 4(b)). With the flow cytometry, we read directly CV values from the software but with the microplate reader, CV values were calculated using the following mathematical equation: CV = 100 × standard deviation/mean, with 6 replicates of each of 10 experiments. There was no difference in the CV values between blockers concentration. Then, we can conclude, the repeatability is independent of the blocker concentrations in the microplate reader. 

**Figure 5:**
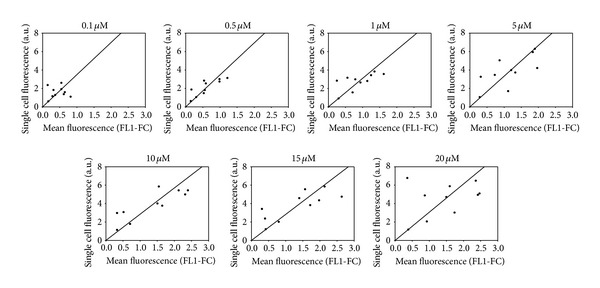
Intermethods comparison of repeatability for increasing PSC833 concentration. Each graph shows the distribution of calcein fluorescence in 10 samples analyzed with both methods. Cell-normalized well fluorescence in microplate reader is expressed as a function of cell fluorescence concentration (FL1-FC) in flow cytometry for increasing concentrations of PSC833. Straight line corresponds to the mean methods fluorescence ratio.

Replicability is the variability of the measurements obtained by one person while measuring the same item repeatedly. To underline the compared repeatability between both methods, the mean fluorescence (FL1-FC) was formulated as function of single cell fluorescence for each PSC833 concentration for the 10 biological replicates (Figure 5). Thus, each point represents a separate experiment within same manipulation condition on MCF-7/Doxo. Each graph represents a PSC833 concentration. For high concentration of PSC833, sample values diverge away from the ratio of the means for the two methods. In conclusion, when blockers are used in high concentration, the single cell fluorescence is more variable.

### 2.6. Difference between the Two Methods

**Figure 6:**
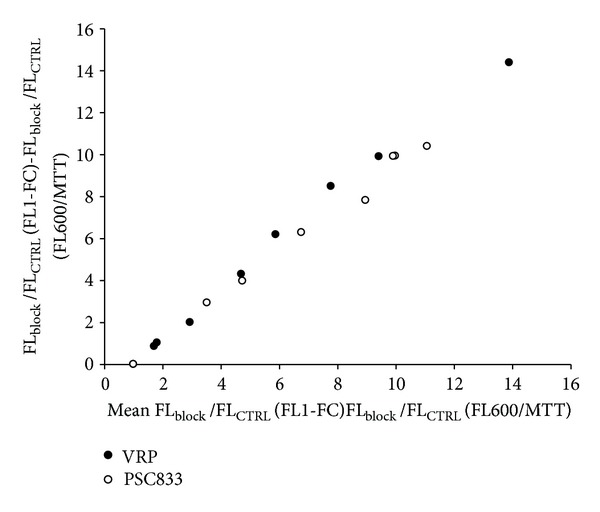
Method responses differential as a function of mean fluorescence. The graph shows the differences between fluorescence measured in flow cytometry (FL1-FC) and microplate reader (cell-normalized well fluorescence) as function of mean fluorescence measured in these two methods. Gap between flow cytometry and microplate reader responses linearly increased with P-gp blockers-induced fluorescence accumulation.

To compare both measuring techniques, we formulated the differences by using Altman and Bland method [21]. Fluorescence was expressed as a function of control (FL_block_/FL_ctrl_ = Δ*F*). Mean of flow cytometry fluorescence or MPR fluorescence were shown on the *x* axis and the difference on the *y* axis (Figure 6). When the blockers concentration was increased, the difference between the two methods was higher. This result means that the difference between the highest fluorescence and the lowest fluorescence is more important in flow cytometry than in the MPR.

### 2.7. Doses Responses Curves

**Figure 7:**
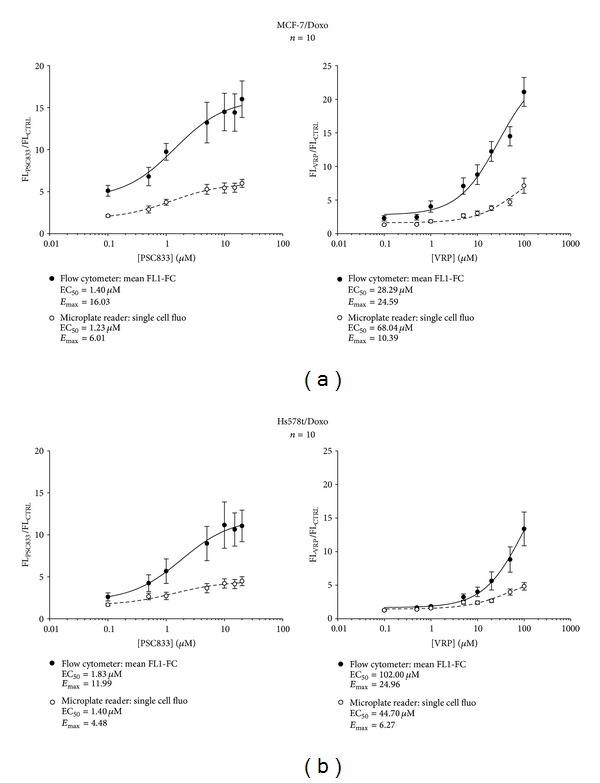
Intermethods comparison of PSC833 and verapamil doses responses curves expressed as mean fluorescence. Superimposed microplate reader and flow cytometry semilogarithmic doses responses curves showing the effect of increasing verapamil or PSC833 concentrations on calcein accumulation in MCF-7/Doxo (a) or Hs578T (b). Each point represents means ± sem (10 independent experiments) of cell-normalized well fluorescence (open circles) or flow cytometry mean FL1-FC (filled circles) expressed as the ratio of signals in the presence of blocker to signals in control conditions. Ligand binding sigmoidal doses responses curves were fitted to the data to obtain blockers potencies (half-maximal effective concentration, EC_50_) and efficacies (maximum response, *E*_max⁡_).

Doses responses curves represented in Figure 7 allowed us to determine the EC_50_ (half maximal effective concentration) of each blocker for each method. The experimental point was fitted with doses-responses curves by Sigma Plot 11 Software. EC_50_ and *E*_max⁡_ (maximal effect) were reported in Table 2. Although, the potency of PSC833 in all cell lines tested was similar with the two methods, results with VRP were highly fluctuating. Based on the differences seen when the blocker concentration increases, we conclude that efficacy level obtained with flow cytometry was higher than that of the microplate reader method.

**Figure 8:**
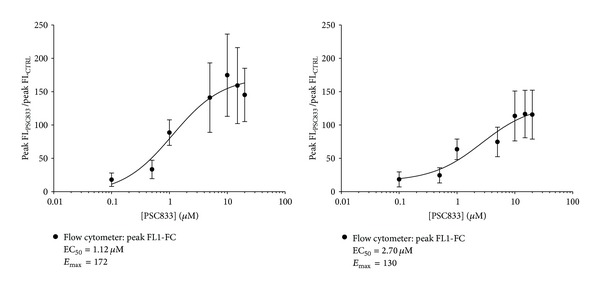
Flow cytometry PSC833 doses responses curves obtained by analyzing modal single cell fluorescence concentration (FL1-FC). Semilogarithmic doses responses curves showing the effect of increasing PSC833 concentrations on calcein accumulation in MCF-7/Doxo. Each point represents peak (modal) ± sem (10 independent experiments) of FL1-FC in MCF7/Doxo (a) or Hs578T/Doxo (b) expressed as the ratio of signal in the presence of PSC833 to signal in control conditions. Ligand binding sigmoidal doses responses curves were fitted to the data to obtain PSC833 potency (half-maximal effective concentration, EC_50_) and efficacy (maximum response, *E*_max⁡_).

Therefore, analysing the mean fluorescence of the population resulted in a loss of information using the technique flow cytometry. Indeed, for the low level of fluorescence, the signal varies from 1 to 10, whereas for the higher one, the signal oscillated between 10 and 5000 (Figure 2). This indicates that at the time of measurement, the inhibitors did not have the same efficacy on the different cell subpopulations. Therefore, when using the inhibitors, the fluorescence mean values were different from the most often detected fluorescence (fluorescence at the peak or mode). Thus, we represented the doses responses curves with the fluorescence at the peak for the PSC833 in flow cytometry (Figure 8). This data representation is impossible for the VRP because this inhibitor was not effective enough. EC_50_ calculated with this representation seem to fit with the data obtained for the mean of fluorescence, while the *E*_max⁡_ values were completely different. 

## 3. Discussion and Conclusion

In this paper, we compared simple, sensitive, and specific methods with fluorescence detection for the P-gp efflux activity in MCF-7 and H5578T, as well as in resistant variants of these cell lines, MCF-7/Doxo and H5578T/Doxo. Regardless of the technical issues, both described methods, flow cytometry and microplate reader can be used to measure the *in vitro* effectiveness of P-gp inhibitors at low concentrations. First, we studied the effects of P-glycoprotein blockers on calcein-AM efflux by quantifying the empire fluorescence of the whole well or cell-normalized well fluorescence with a microplate reader. There were no important differences between the two different ways of analysis, however, we should emphasize that we used two MDR cell lines that are really homogenous. The same experiments should be performed using coculture or primary cells. This conclusion is the same for the analysis with FL1 and FL1-FC in flow cytometry. When we compare the results obtained with flow cytometry or MPR, we can see that the differences between basal fluorescence and fluorescence in presence of blockers are nearly the same between the two methods. Both methods have the same range of scale to detect P-gp activities. And this is confirmed by the LOD analysis.

The intramethod comparisons showed that when we analysed the fluorescence by flow cytometry the CV value is inversely correlated with the fluorescence level, which correlates to the efflux activity. Therefore, the flow cytometer is more accurate for higher level of inhibition. When we analysed the fluorescence by MPR, the CV values seem to be independent of the efflux activity. We could therefore discern a “V” pattern using the MPR CV values, with the smaller CV values reflecting intermediate efflux activity. 

The two methods indicate with precision the blocker answers disparity. Thereby, when we express the single cell fluorescence in MPR as a function of the mean FL1-FC, we can notice that when the fluorescence increases, the values are dispersing around the diagonal but remain around the medium ratio values.

When we compared the two techniques by using the Altman and Bland method, we noticed that when the fluorescence increases the difference between the two methods was amplified. The fluorescence variance increases linearly with the fluorescence level which suggests that the efficacy of the MPR decreases with the increasing of P-gp inhibition. Thus, we were not able to determine an accurate value for *E*_max⁡_ with the MPR method. There is also a consequence on the EC_50_ determination. For the more effective inhibitor (PSC833), EC_50_ is the same with the two methods. However, for the less effective inhibitor (VRP), the curve never reaches saturation and as a result of that, the accurate *E*_max⁡_ cannot be determined by the MPR technique. Using flow cytometry, PSC833 doses responses curves obtained by analysing modal single cell fluorescence concentration (FL1-FC) confirmed the EC_50_ values obtained with the classical doses responses curve. Therefore, the *E*_max⁡_ values are higher because no detection of the fluorescence dilution was detected from the cells which are less affected by the inhibitor.

In conclusion, the MPR is more powerful for the detection of small inhibition but the flow cytometry method is more reliable at higher concentrations of inhibitors. This observation is the same for both inhibitors tested. Therefore, it is independent of the sort of the blocker. To determine precisely the inhibition efficacy it is important to use the flow cytometer. To give correct value for *E*_max⁡_ and EC_50_, we cannot rely on the MPR method.

Therefore, microplate reader could be used to screen P-gp blockers and flow cytometry for accurate analysis. In summary, the presented methods are simple and can be routinely used in determination of potential P-gp inhibition of various compounds. Depending on the user's specific needs, both methods can be applicable and potentially used to detect effects of competitive and noncompetitive P-gp blockers.

## 4. Methods

### 4.1. Cell Lines

The study was carried out with human breast carcinoma derived cells, MCF-7 and Hs578T and multidrug resistant variant of these cells lines (MCF-7/DOXO and Hs578T/DOXO), kindly obtained from Pr. J. P. Marie (Hôtel Dieu, Paris, France). MCF7/DOXO and Hs578T/DOXO cells were, respectively, isolated by stepwise selection with increasing concentrations of doxorubicin [22]. Cells were maintained in RPMI 1640 (Sigma, St. Louis, MO) containing 5% heat-inactivated fetal bovine serum (Sigma, St. Louis, MO), 2 mM L-glutamine (Sigma, St. Louis, MO), and 1% antibiotic/antimycotic solution (Sigma, St. Louis, MO) and incubated in a humidified atmosphere containing 5% of CO_2_ at 37°C.

### 4.2. Reagents

Purified doxorubicin (DOXO), verapamil (VRP), DMSO, MTT (3-(4,5-dimethylthiazol-2-yl)-2,5-diphenyltetrazoliumbromide), and phosphate buffer saline (PBS buffer, pH 7.4) were purchased from Sigma (St. Louis, MO). Calcein acetoxy-methylester (calcéine-AM) was supplied by Invitrogen Life Technologies (Carlsbad, CA). SDZ PSC833 (PSC833) was kindly provided by Pr. J. P. Marie (Hôtel Dieu, Paris, France). Final concentration of DMSO and H20 was less than 0.1%. 

### 4.3. Samples Preparation

For the calcein-AM efflux assay, cells were cultured at 80 or 90% confluency in T25. Cells were detached by trypsin/EDTA treatment. The experiments were initiated by washing the cells with PBS. Cells were treated with 0, 1, or 10 *μ*M of antagonists VRP or PSC833 at 37°C for 30 min. Then 0,25 *μ*M of calcein-AM were added to each well. After 15 min incubation at 37°C, cells were washed twice with PBS and splitted to quantify P-gp activity by fluorescence in a microplate reader or in a flow cytometer. Ten experiments were independently performed for each condition. 

### 4.4. Measurement of P-gp Activity

For the evaluation activity by flow cytometry, fluorescent light (FL) was quantified using a cell lab quanta SC MPL flow cytometer (Beckman Coulter) equipped with a 22 mW 488 nm excitation laser. The voltage settings of photomultipliers remained unchanged throughout the experiments. For each analysis we recorded 10,000 events, triggered on electronic volume (EV) as primary parameter, according to a particle diameter exceeding 8 *μ*m. Green FL of calcein was quantified via the FL1 channel (log scale) through a 525 nm band pass filter. 

For the evaluation of P-gp activity by microplate reader, calcein fluorescence was quantified using a FL-600 microplate fluorometer (Bio-tek instruments, Winooski, VE). For each experiment, six wells were measured in parallel (*k*_ex_ = 494 nm and *k*_em_ = 517 nm). 

### 4.5. Cell Viability Study (MTT Assay)

In order to take the number and viability of cells into account, an MTT assay was systematically performed after reading the calcein fluorescence. 10% of MTT reagent was added to each well to a final concentration of 500 *μ*g/mL, and the cells were incubated for 4 hours at 37°C. The medium has been then replaced with 200 *μ*L of DMSO to dissolve the reaction product. The optical density was read at 570 nm versus 630 with an Elx 808 microplate reader (Bio-tek Instruments, Winooski, VE). Normalized calcein accumulation was then expressed as the ratio of the well fluorescence to the MTT absorbance.

### 4.6. Statistics

For statistical analysis and graphical representation, Excel (Microsoft Corporation) and SigmaPlot (SysStat, Erkrath, Germany) softwares were used. Numerical results are given as means ± SEM (*n* = sample size). The statistical significance was assessed with SigmaPlot or Excel software according to either a Student's *t*-test. Statistical significance was accepted for *P < 0.05; **P < 0.01; ***P < 0.001.

## Figures and Tables

**Table 1:** Intermethods comparison of fluorescence increase detection thresholds.

	MCF-7		Hs578T	
	0,1 *µ*M VRP	0,1 *µ*M PSC833	0,1 *µ*M VRP	0,1 *µ*M PSC833
*Flow cytometer *				
Mean FL1	∗∗	∗∗∗	∗	∗∗∗
Mean FL1-FC	∗∗	∗∗∗	∗	∗∗
*Microplate reader *				
Well fluorescence	∗∗∗	∗	∗∗	∗∗
Single cell fluorescence	∗∗∗	∗∗∗	∗∗	∗∗

Flow cytometer (mean FL1 and mean FL1-FC) and microplate reader (whole well fluorescence and mean single cell fluorescence) signal increase detection thresholds were estimated by testing groups of untreated cells and cells treated with the lowest P-gp antagonists concentration (0,1 *µ*M) for significant differences (*P < 0,05; **P < 0,01; ***P < 0,001; paired Student's *t*-test; *n* = 10).

**Table 2:** EC_50_ and *E*_max⁡_ values obtained from the doses responses curves.

	EC_50_ (*µ*M)		*E*_max⁡_	
	FC	MPR	FC	MPR
MCF-7/Doxo PSC833	1,40	1,23	16,03	6,01
MCF-7/Doxo VRP	28,29	68,04	24,59	10,39
Hs578T/Doxo PSC833	1,83	1,40	11,99	4,48
Hs578T/Doxo VRP	102,00	24,96	44,70	6,27

Values are given for the two methods (flow cytometry—FC and microplate reader—MPR) with the two different antagonists for the two cells lines, MCF-7/Doxo and Hs578T.
